# Addressing opioid use disorder among rural pregnant and postpartum women: a study protocol

**DOI:** 10.1186/s13722-020-00206-6

**Published:** 2020-10-31

**Authors:** M. Aryana Bryan, Marcela C. Smid, Melissa Cheng, Katherine T. Fortenberry, Amy Kenney, Bhanu Muniyappa, Danielle Pendergrass, Adam J. Gordon, Gerald Cochran

**Affiliations:** 1 Program for Addiction Research, Clinical Care, Knowledge, and Advocacy (PARCKA), Division of Epidemiology, University of Utah School of Medicine, 295 Chipeta Way, Salt Lake City, UT 84132 USA; 2 Department of Obstetrics and Gynecology, University of Utah School of Medicine, Division of Maternal Fetal Medicine, Salt Lake City, UT 84132 USA; 3 Department of Family and Preventive Medicine, University of Utah School of Medicine, 375 Chipeta Way Ste. A, Salt Lake City, UT 84108 USA; 4 Department of Pediatrics, University of Utah School of Medicine, Division of General Pediatrics, 295 Chipeta Way, Salt Lake City, UT 84108 USA; 5 Eastern Utah Women’s Health, LLC, 77 S 600 E, Price, UT 84501 USA; 6 Greater Intermountain Node (GIN), NIH NIDA Clinical Trials Network, University of Utah, Salt Lake City, UT USA; 7 Informatics, Decision-Enhancement, and Analytic Sciences Center, VA Salt Lake City Health Care System, Salt Lake City, UT USA

**Keywords:** Perinatal addiction, Medication-assisted treatment, Medication for opioid use disorder, Rural, Opioid use disorder, Provider education

## Abstract

**Background:**

Opioid use disorder (OUD) among women delivering at a hospital has increased 400% from 1999–2014 in the United States. From the years 2007 to 2016, opioid-related mortality during pregnancy increased over 200%, and drug-overdose deaths made up nearly 10% of all pregnancy-associated mortality in 2016 in the US. Disproportionately higher rates of neonatal opioid withdrawal syndrome (NOWS) have been reported in rural areas of the country, suggesting that perinatal OUD is a pressing issue among these communities. There is an urgent need for comprehensive, evidence-based treatment services for pregnant women experiencing OUD. The purpose of this article is to describe a study protocol aimed at developing and evaluating a perinatal OUD curriculum, enhancing evidence-based perinatal OUD treatment in a rural setting, and evaluating the implementation of such collaborative care for perinatal OUD.

**Methods:**

This two-year study employed a one group, repeated measures, hybrid type-1 effectiveness-implementation design. This study delivered interventions at 2 levels, both targeting improvement of care for pregnant women with OUD. The first area of focus was at the *community healthcare provider-level*, which aimed to evaluate the acceptability and feasibility of perinatal OUD education across time and to improve provider education by increasing knowledge specific to: MOUD provision; screening, brief intervention, and referral to treatment (SBIRT) utilization; and NOWS treatment. The second area of intervention focus was at the *patient-level*, which assessed the preliminary effect of perinatal OUD provider education in promoting illicit opioid abstinence and treatment engagement among pregnant women with OUD. We adopted constructs from the Consolidated Framework for Implementation Research (CFIR) to assess contextual factors that may influence implementation, and the Reach, Effectiveness, Adoption, Implementation, and Maintenance (RE-AIM) model to comprehensively evaluate implementation outcomes.

**Discussion:**

This article presents the protocol of an implementation study that is employing the CFIR and RE-AIM frameworks to implement and evaluate a perinatal OUD education and service coordination program in two rural counties. This protocol could serve as a model for clinicians and researchers seeking to implement improvements in perinatal care for women with OUD in other rural communities.

*Trial registration* NCT04448015 clinicaltrials.gov.

## Background

Opioid use disorder (OUD) among women delivering at a hospital has increased 400% from 1999–2014 in the United States [1] From the years 2007 to 2016, opioid-related mortality during pregnancy increased over 200%, and drug-overdose deaths made up nearly 10% of all pregnancy-associated mortality in 2016 in the US [2]. Access to care and lack of provider understanding of evidence-based treatment for perinatal OUD are both contributing factors to drug-related pregnancy-associated and pregnancy-related deaths, particularly in rural areas [3, 4]. Disproportionately high occurrences of neonatal opioid withdrawal syndrome (NOWS) have been reported in rural areas of the country, suggesting that perinatal OUD is a critical issue among rural communities [5].

Without treatment, maternal OUD is associated with adverse perinatal outcomes including increased risk of preterm delivery, low birth weight and difficulty breastfeeding [6–10] Undertreatment of maternal OUD also increases risk of maternal return to use, overdose and death [3, 11]. Effective treatment is available for maternal OUD during pregnancy—specifically medication for opioid use disorder (MOUD) and psychosocial services [12]. Maternal treatment for OUD is associated with significant benefits to both the mother and infant [12–15] There is an urgent need for comprehensive, evidence-based OUD treatment services specifically for pregnant women experiencing this disorder. OUD is a chronic disease and will persist beyond pregnancy; the focus of this study is addressing the specific treatment gap evident for women experiencing simultaneous OUD while pregnant and/or postpartum (i.e. perinatal OUD).

### Barriers to implementation in rural areas

Rates of maternal opioid use and NOWS in rural areas have climbed substantially over the past ten years compared to their urban counterparts [16], yet treatment engagement among this population has failed to keep pace [17], While the effectiveness of MOUD in treating OUD is well-established, [13] the limited availability of buprenorphine (BUP) prescribers and methadone clinics in rural areas has created a substantial barrier to accessing these life-saving treatments for those with OUD—including pregnant women [18, 19]. Within rural areas, buprenorphine prescribers frequently do not treat pregnant patients [20]. Indeed, mothers with OUD and their infants living in rural areas frequently require transfer to other hospitals following delivery to receive high-level, specialty substance use and neonatal care [21]. Perceptions of OUD as a moral failing among pregnant women have given rise to punitive approaches rather than evidence-based medical care [22]. Rural prescribers also identify stigma from other providers as a major barrier to providing MOUD [23]. Lack of specialty support for complex problems, such as perinatal OUD, also creates a significant barrier to rural healthcare providers offering MOUD [24]. As of 2017, 60.1% of rural counties did not have a buprenorphine-waivered physician [25].

Substantial gaps exist for pregnant women with OUD receiving evidence-based care, and this is particularly true in rural America. Presently, little research is available regarding implementation of evidence-based services for these women. It is critical to identify replicable and sustainable approaches to implement needed OUD care for this vulnerable population. This article presents the protocol of an implementation study that is employing the Consolidated Framework for Implementation Research (CFIR) [26] and the Reach, Effectiveness, Adoption, Implementation, and Maintenance (RE-AIM) [27] framework to implement and evaluate a perinatal OUD education and service coordination program in two rural Utah counties. The CFIR serves to assess contextual factors at the beginning and throughout the project that may influence implementation success, while the RE-AIM functions as a post-implementation framework to assess implementation outcomes. This protocol could serve as a model for clinicians and researchers seeking to implement improvements in perinatal care for women with OUD in rural communities. The purpose of this project is to describe a study protocol aimed at developing and evaluating a perinatal OUD curriculum, enhancing evidence-based perinatal OUD treatment in a rural setting, and evaluating the implementation of such collaborative care for perinatal OUD.

## Methods

### Design

This two-year study is employing a one group, repeated measures, hybrid type-1 effectiveness-implementation design, [28] which emphasizes clinical effectiveness monitoring while also gathering information regarding the implementation of the program. This study delivers interventions at 2 levels, both targeting improvement of care for pregnant women with OUD. The first area of focus is at the *community healthcare provider-level*, which aims to evaluate the acceptability and feasibility of perinatal OUD education across time and to improve provider education by increasing knowledge specific to: MOUD provision; screening, brief intervention, and referral to treatment (SBIRT) utilization; and NOWS treatment. The second area of intervention focus is at the *patient-level*, which assesses the preliminary effect of perinatal OUD provider education in promoting illicit opioid abstinence and treatment engagement among pregnant women with OUD. The research team is interested in assessing changes across time among recipients. Time and resources necessary to implement an experimental or quasi-experimental design are not available for this project. For this reason, the research team has chosen to utilize a single arm design. The University of Utah Institutional Review Board approved this study.

### Study setting

The study setting for this project is rural Utah. From 1999–2014, the proportion of women with OUD delivering at a hospital in Utah has increased nearly ten-fold [1]. Utah ranks among the highest nationally in terms of opioid overdose rates among women [29] and frequency of opioid prescribing to pregnant women insured by Medicaid (41.6%) [30]. The leading cause of deaths among pregnant and postpartum women in this state is drug-related, with 77% of these deaths involving opioids [3].

Two adjacent rural counties [31] in Utah were chosen as the target for the intervention, with a local women’s health clinic as the focal point of care. These counties were selected because of the high rates of opioid overdose, limited resources, and high degree of existing community engagement [32–34]. These two rural counties lead the state in rates of opioid overdose deaths, with collectively 47.7 per 100,000 compared to the state average of 17.4 deaths per 100,000 [32].

### Implementation framework

In order to accomplish our objectives, we are adopting constructs from the CFIR to assess contextual factors that may influence implementation [26]. We also are utilizing the RE-AIM model to efficiently and comprehensively evaluate implementation outcomes of the multilevel intervention [27].

Five domains characterize the CFIR: *intervention characteristics, outer setting, inner setting, characteristics of the individuals involved*, and *the process of implementation*. Within each domain, we have identified several constructs relating to the efficacy, sustainability and reproducibility of the intervention, and are using these constructs to guide the development and execution of the project (Table 1). We are assessing *intervention characteristics,* such as evidence strength and quality, complexity of the intervention, and relative advantage of the intervention to help guide the development and dissemination of our perinatal OUD education curriculum. Further, within the *inner setting*, the research team is examining structural characteristics at the healthcare provider level in order to improve our understanding of the climate and characteristics of the organizations that would be receiving the intervention. Also related to the *inner setting*, we are assessing *characteristics of individuals involved*, such as knowledge and beliefs about the intervention and individual stage of change, to inform the research team on how to make the education information more appealing to providers and support uptake by providers and patients. We also are engaging with community representatives to gain knowledge about *outer setting* components of the CFIR model, such as cosmopolitanism, in order to understand the degree to which local organizations are linked and work together. We are also working to assess patient needs and resources, as well as to understand knowledge of providers regarding barriers and facilitators for meeting patient needs. Finally, the *process of implementation* domain is guiding our understanding of the efforts of implementation leaders and champions during implementation preparation with respect to planning and engaging in the project.

**Table 1:** Consolidated framework for implementation research targeted domains at the healthcare provider, patient advocate, and community representative levels

CFIR domain	CFIR construct	Level
Intervention characteristics	Evidence strength and quality	Healthcare provider level
	Complexity	Healthcare provider Patient advocate
	Relative advantage	Healthcare provider Patient advocate
Outer setting	Cosmopolitanism	Community Representative
	Peer pressure	Community Representative
	Patient needs and resources	Community Representative
Inner setting	Structural characteristics	Healthcare provider
	Readiness for implementation	Healthcare provider
	Implementation climate	Healthcare provider
Characteristics of individuals involved	Knowledge and beliefs about the intervention	Healthcare provider Patient Advocate
	Individual stage of change	Healthcare provider Patient advocate
Process of implementation	Planning	Healthcare provider Community representative Patient advocate
	Engaging	Healthcare provider Community representative Patient advocate
	Formally appointed internal implementation leaders	Healthcare provider
	Champions	Healthcare provider Patient advocate

The RE-AIM model is an effective tool for comprehensively evaluating multilevel interventions using five dimensions [27] (Table 2). Using this framework provides a streamlined way to assess the various components of an implementation project and the intervention itself, allowing our team to understand which components are facilitators or barriers of its overall success in a real-world environment. Specifically, we will use the *Reach* and *Adoption* aspects of this framework to assess uptake of the intervention, as operationalized in Table 2. We further will rely on the *Efficacy*, *Implementation,* and *Maintenance* components of RE-AIM to evaluate the success of the intervention and implementation strategy in educating healthcare providers over the course of the study and afterward, as described in Table 2. This evaluation approach maps on well with the hybrid effectiveness-implementation study design [27, 28].

**Table 2:** RE-AIM Evaluation of Perinatal Addiction Education

Dimension and questions addressed	Data sources	Methods	Outcome/Process measures
Reach			
To what extent did perinatal addiction education reach the providers treating pregnant women with OUD in Your Counties?	Department of Professional Licensing	Identify all healthcare providers in the community licensed to potentially treat pregnant women with OUD	Number of community providers included in the study/ total number of community providers working with pregnant women with OUD
To what extent did perinatal addiction education reach the pregnant women with OUD within the healthcare system in Your Counties?	Local healthcare and social service agencies	Electronic medical records; health department records	Number of patient participants/ total number of pregnant women with opioid misuse in Your Counties during the study duration
Efficacy			
How effective was the provider training and support?	Community providers	Questionnaires assessing skills, knowledge and satisfaction	Mixed method surveys regarding provider experience and skillset
How effective were perinatal addiction education services in improving care for pregnant women with OUD?	Healthcare and social service records; patient participants	Compare data regarding illicit substance use, MOUD continuity, opioid poisoning occurrences, physical and mental health, and social service involvement; questionnaires to assess patient perception	Medical codes; standardized comprehensive wellness measurement instruments; qualitative surveys
Adoption			
To what extent are community providers using perinatal addiction education practices when treating patients with perinatal OUD?	Community providers; local healthcare agencies	Questionnaires assessing provider practices and attitudes; electronic medical records	Mixed methods surveys regarding provider practices; frequency of MOUD provision, NOWS treatment and SBIRT by participating community providers
To what extent are patients utilizing resources made available through the perinatal addiction education program?	Partnering agencies (e.g. EUWH, Four Corners Behavioral Health); medical records; pharmacy records	Interviews with partner agencies; electronic medical record and pharmacy record evaluation	Utilization of care management and resources made available through this project; number of MOUD prescriptions filled; presence of BUP in urinalysis via medical record
Implementation			
What were the barriers and facilitators of high quality care?	Community providers	Questionnaires and focus groups	Perceptions of and feedback regarding the implementation kickoff event, as well as ongoing training and support provided via the implementation of perinatal addiction education
Maintenance			
Will the perinatal addiction education practices be maintained over time in Your Counties?	Community providers; healthcare administrators	Interviews and questionnaires with community providers and healthcare administrators regarding provider practices and policies	Questionnaire regarding how integrated perinatal addiction education practices are into standard treatment of pregnant women with OUD and what policies exist to support them

*OUD* Opioid Use Disorder, *MOUD* Medication for Opioid Use Disorder, *NOWS*  Neonatal Opioid Withdrawal Syndrome, *SBIRT* Screening, Brief Intervention, and Referral to Treatment; *BUP* Buprenorphine

### Participants

Study participants are comprised of [1] community health-care providers and [2] pregnant women with OUD. Community health-care providers include nurse practitioners, physician’s assistants, obstetricians, pediatricians, family practice physicians, behavioral health care professionals, and child protective services caseworkers practicing within the targeted counties who are willing to participate in the educational sessions delivered by the project team and provide informed consent.

Pregnant women with OUD participants include those who are living in the targeted counties, who are not currently incarcerated, and who provide informed consent. During the intervention phase, women are encouraged to engage in in physical, mental, or behavioral health service by the project nurse care manager that is congruent with the participants needs for prenatal care, OUD recovery, and wellness for mother and fetus. These participants are being recruited with the help of the community-based nurse care manager through posting fliers and communicating directly with potential referral sources, such as local healthcare providers, local health department, and the division of child and family services. The nurse care manager also obtains informed consent. Although the study sample is not powered a priori to achieve a pre-specific treatment effect, a nurse care manager who is well integrated in the local community is part of the research team and leverages relationships with local referral sources to achieve adequate participant enrollment. The nurse care manager obtains locator information for each enrolled participant, and continues checking in with participants to ensure retention and follow-up. The research team meets weekly with the nurse care manager to ensure several follow-up attempts are made for purposes of enrolling and collecting data from participants.

### Needs assessment and implementation evaluation

The CFIR and RE-AIM models are being used in complement to one another to guide and evaluate implementation of this project. The CFIR is used in this study to assess contextual factors influencing implementation in the needs assessment and throughout study duration to assess continued needs and any changes across time related to the project interventions [26]. The RE-AIM model is applied to efficiently and comprehensively evaluate implementation outcomes of the multilevel intervention at the end of the study [27].

Prior to the implementation of the educational intervention, the research team conducted a community needs assessment. This was done in order to measure CFIR constructs that may influence implementation success (Table 1), and included healthcare provider, community representative, and patient advocate assessments (see Additional file 1: Figs. S1–3). Likewise, this study also includes assessments at these three levels at the study midpoint and following study conclusion, which have yet to be completed in real-time. In real-time, pre-implementation components of this project have been completed. Data collection at the patient-level begins when participants enroll in the study and continues through the end of their study participation. At study conclusion, RE-AIM measures of implementation success are evaluated (Table 2). Finally, analyses occur at the conclusion of the study after patient enrollment has ended.

#### Windshield survey

The pre-implementation needs assessment also included a windshield survey to help researchers gain an understanding of the community characteristics [35]. Windshield surveys involve surveying an area of interest by foot or in a vehicle and recording observations related to community safety, resources, barriers, and accessibility of care. This project component followed a standardized guide adapted from Callan, [35] wherein trained research assistants drove within areas of the targeted counties where health care services are primarily located to assess clinic/agency availability, location and distance, physical accessibility (sidewalks, bus stops), and presentation (graffiti, general appearance, disrepair). Results of the windshield survey allowed our team to identify service gaps and needs within the targeted communities and surrounding areas.

#### Provider assessments

Provider assessments occur at three distinct time-points: pre-implementation (immediately preceding the kick-off event), mid-implementation (3 monthspost-kick-offevent), and post-implementation (final month of the project) (see Table 3). Qualitative interview questions were adapted from the CFIR Interview Guide Tool [36]. In the pre-implementation phase, these interviews informed our needs assessment, and discussed barriers and facilitators with retention of women with OUD in obstetric and OUD care and provider skills and knowledge in training topic areas. In the mid- and post-implementation phases, qualitative items were modified to assess any improvements related to the intervention implementation as well as provider satisfaction with support/education (see Table 2).

**Table 3:** Provider-Level Study Measures

Outcome	Level	Domain	Instrument	Source	Pre-Implementation (Time 1)	Mid-Implementation (Time 2)	Post-Implementation (Time 3)
Patient retention in care	Provider	QI	# patients treated/provider	Self-report	X	X	X
Pre-/Post-learning knowledge & skills (physical health)	Provider	QI	Mixed methods survey	Self-report	X		
Pre-/Post-learning knowledge & skills (mental health)	Provider	QI	Mixed methods survey	Self-report	X		
Provider satisfaction with support	Provider	QI	Mixed methods survey; quantitative interview	Self-report	X	X	X
Provider attitudes and perceptions towards substance misuse	Provider	QI	SAP-1; AAPPQ/adapted AAPPQ	Self-report	X	X	X
Acceptability of intervention	Provider	QI	EBPAS	Self-report	X	X	X
Perception of improvement in care for pregnant women with OUD in community	Provider	QI	Mixed methods survey; quantitative interview	Self-report			X
Program Maintenance	Provider	QI	Mixed methods survey; quantitative interview	Self-report			X
Adoption of evidence-based practices	Provider	QI	Mixed methods surveys; quantitative interview	Self-report		X	X

SAP-1 SBIRT Attitudes & Perceptions, *AAPPQ* Alcohol & Alcohol Problems Perceptions Questionnaire, *EBPAS* Evidence-Based Practices and Attitudes Scale

Assessments also included self-report quantitative assessments administered via web-based survey. Pre-implementation quantitative surveys also helped to inform our needs assessment. These surveys were adapted from the Evidence-Based Practices and Attitudes Scale (EBPAS) which assesses provider attitudes and perceptions towards adopting new interventions, [37] and the SBIRT Attitudes and Perceptions (SAP-1) Questionnaire which assesses substance use screening, intervention, and referral services competency [38]. Additionally, the Alcohol and Alcohol Problems Perceptions Questionnaire (AAPPQ) was utilized and adapted for opioid use to assess provider attitudes towards serving individuals with substance use [39]. In the mid- and post-implementation phases, quantitative items were repeated in a modified form to assess any improvements related to the intervention implementation. In addition, providers participated in the baseline kick off training which included a webinar on perinatal OUD treatment and NOWS presented by research team physicians. Attendees completed learning assessments related to the training sessions and subsequent satisfaction.

#### Participant assessments

Women who have provided consent are asked to complete a baseline assessment prior to receiving the intervention. This assessment consists of a series of self-report and interviewer administered questionnaires regarding health, mental health, social service involvement, and substance use. These constructs were selected for assessment given that the interventions and services provided to participants were anticipated to have an impact on these areas. Two follow-up assessments are collected: a pre-delivery follow-up at 34 to 40 weeks of gestation and a post-delivery follow-up 30 days following delivery. These assessments contain the same questionnaires as the baseline assessment, with the exception of the Adverse Childhood Experience scale since these scores are related to childhood experiences and therefore should not vary over the course of the study. We are also collecting data from the woman’s and her infant’s medical records (Table 4). In addition, our team will work with the local department of health to obtain county level data across time at the conclusion of the project to assess intervention impact within the larger community (Table 4). In the event that a participant chooses to discontinue their participation in the study, no additional data will be collected from that participant.

**Table 4:** Patient-level study measures

Source	Outcome	Domain	Instrument	Source of data	Baseline	Pre-delivery follow-up	Post-delivery follow-up
Health department	Hepatitis B Virus (HBV)	Health	ICD10 Code B16	Health department	X		X
	Hepatitis C Virus (HCV)	Health	ICD10 Code B18.2	Health department	X		X
	Human Immunodeficiency Virus (HIV)	Health	ICD10 Code B20	Health department	X		X
	Syphilis	Health	ICD10 Code A53.9	Health department	X		X
	Gonorrhea	Health	ICD10 Code A54.9	Health department	X		X
	Chlamydia	Health	ICD10 Code A74.9	Health department	X		X
	Child welfare involvement	Social	ICD10 Code Z62.21	Health department	X		X
	Overdose	Health	ICD10 Cod T40.6	Health department	X		X
Medical record	Pre-eclampsia	Health	ICD10 Code O14	Medical record			X
	Pre-term birth	Health	ICD10 Code O60	Medical record			X
	Cesarean section	Health	ICD10 Code O82;	Medical record			X
	Intrauterine growth restriction	Health	ICD10 Code P05.9	Medical record			X
	Birth weight	Health	ICD10 Code P07	Medical record			X
	Apgar score	Health	ICD10 Code P09	Medical record			X
	Neonatal opioid withdrawal syndrome (NOWS) severity	Health	ICD10 Code P96.1	Medical record			X
	Length of hospital stay	Health	Discharge dates	Medical record			X
	Continuity of MOUD	QI	Prescriptions Filled	Medical record			X
	Illicit drug use	SUD	Urinalysis	Medical record	X	X	X
	Number of infant ER visits	Health	CPT 99,281–99,285	Medical record			X
	Adequacy of prenatal care	QI	Adequacy of Prenatal Care Utilization (APNCU)	Calculated from medical record			X
Medical record/self-report	Overdose events	SUD	ICD10 Code T40.2X1A	Medical record/Self-report	X		X
Self-report	Overall health	All	12-Item Short Form Survey (SF-12)	Self-report	X	X	X
	Substance misuse	SUD	Adapted Drug Abuse Screening Test (DAST)	Self-report	X	X	X
	Trauma history	Social	Adverse Childhood Experience (ACE)	Self-report	X		
	Alcohol misuse	SUD	Alcohol Use Disorders Identification-Concise (AUDIT-C)	Self-report	X	X	X
	OUD	SUD	Diagnostic and Statistical Manual 5 (DSM-5) Opioid Use Disorder (OUD) Checklist	Self-report	X	X	X
	Depression	Mental Health	Edinburgh Postnatal Depression Scale (EPDS), Patient Health Questionnaire-9 (PHQ-9)	Self-report	X	X	X
	Nicotine dependence	SUD	Fagerstrom Test for Nicotine Dependence (FTND)	Self-report	X	X	X
	Anxiety	Mental health	Generalized Anxiety Disorder-7 (GAD-7)	Self-report	X	X	X
	Psychological distress	Mental health	Kessler Psychological Distress Scale (K6)	Self-report	X	X	X
	Parental knowledge & skills	Social	Parenting and Family Adjustment Scale (PAFAS)	Self-report	X		X
	Patient satisfaction	QI	Patient Satisfaction Questionnaire Short Form (PSQ-18)	Self-report		X	X
	Patient utilization of services	QI	Treatment Services Review-6 (TSR-6)	Self-report	X	X	X
	Post-traumatic stress disorder (PTSD)	Mental health	Primary Care PTSD Screen for DSM-5 (PC-PTSD-5)	Self-report	X	X	X

*QI* quality improvement, *SUD* Substance Use Disorder, *IC10* International Classification of Disease, 10th Revision, *CPT* Current Procedural Terminology

Our team chose several reliable and valid self-report instruments to assess substance use at the patient-level. The Alcohol Use Disorders Identification-Concise (AUDIT-C) is a measure of unhealthy alcohol use [40]. The Diagnostic and Statistical Manual 5 (DSM-5) OUD Checklist is an instrument to assess OUD [41]. The Fagerstrom Test for Nicotine Dependence (FTND) captures the presence of nicotine dependence [42]. Finally, we adapted the Drug Abuse Screening Test (DAST) [43] to assess opioid-specific participant behaviors.

To assess mental health, patients complete the Edinburgh Postnatal Depression Scale (EPDS), a 10-item scale for detection of postnatal depression, [44] as well as the Patient Health Questionnaire-9 (PHQ-9) [45]. Participants also complete the Generalized Anxiety Disorder-7 (GAD-7) to assess anxiety, [46] and the Kessler Psychological Distress Scale (K6) to capture psychological distress [47]. We also administer the 12-Item Short Form Survey (SF12) to assess overall well-being and health functioning [48]. Participants likewise complete the Adverse Childhood Experience (ACE) questionnaire, [49] a 10-item instrument inquiring about the presence of childhood abuse and household dysfunction, as well as the 5-item Primary Care PTSD Screen for DSM-5 (PC-PTSD-5) [50] for detecting the presence of post-traumatic stress disorder (PTSD) in a healthcare setting. We also utilize the 30-item Parenting and Family Adjustment Scale (PAFAS) [51] to assesses parenting practices and family relationships.

Lastly, our team also assesses participant satisfaction with the nurse care manager intervention and utilization of services. To assess this area, we administer the Patient Satisfaction Questionnaire Short Form (PSQ-18) [52] and the Treatment Services Review-6 (TSR-6) which captures utilization of healthcare services such as care management [53]. Additionally, we utilize a care management output form, which captures the frequency and type of patient outreach completed by the nurse care manager weekly, including number of calls, texts, greeting cards, accompanied appointments, linkage to psychosocial support, and referrals to medical provider.

Outcomes for women and neonates obtained from medical record assessments include pre-eclampsia, pre-term birth, cesarean section, intrauterine growth restriction, birth weight, Apgar score, NOWS severity, maternal and infant length of hospital stay, continuity of MOUD, illicit drug use, number of infant ER visits, and the Adequacy of Prenatal Care Utilization (APNCU) index is calculated to measure resource utilization [48]. NOWS severity will be assessed using the modified Finnegan Neonatal Abstinence Scoring System, [54] as this is the method utilized by the local hospital that serves the target counties. Data to be collected from local health officials at the conclusion of the study will include county-level prevalence of hepatitis B virus, hepatitis C virus, human immunodeficiency virus (HIV), gonorrhea, chlamydia, number of overdoses, and child welfare involvement.

### Interventions

#### Provider education

Provider education initiated with introductory webinar lectures from the team’s clinical educators and was delivered to the community providers in rural Utah, with one session in person and then repeated via webinar. This introductory presentation was led by perinatal OUD and pediatric specialists and included an introduction to the topic of OUD among pregnant women, the impact of OUD treatment on maternal health, and an overview of MOUD dosage and practice in relation to NOWS. Ongoing educational sessions are being held monthly using videoconferencing, allowing for case consultation and additional training developed by perinatal OUD and pediatric specialists. For those in the area who cannot attend the monthly webinars, local project partners attended staff meetings or have one-on-one visits to deliver the materials and answer questions. Furthermore, all sessions are recorded and posted on the project YouTube channel (with case consultation discussion not included). Sessions have included topics such as, but not limited to, effective use of urine testing, warm handoffs and referrals, peer support services, and substance use stigma. Furthermore, nursing and medical staff of local clinics and child protective service workers have also been trained in SBIRT using both in person and online modalities.

#### Resource enrichment

In addition to improved provider knowledge and skills through the education portion of this intervention, women who participate in this study receive enriched perinatal care. A nurse care manager hired through the project and embedded in the local women’s health clinic provides care coordination for participants. Nurse care management services involve activities such as regular follow-up with enrolled women between appointments in order to promote ongoing engagement in care; resource identification, referral, and enrollment assistance with relevant resources, such as mental health care, support groups, and social services.

### Analyses

Following study conclusion, we will employ descriptive statistics to calculate measures of central tendency, frequencies, and proportions for patient demographics and substance use, health, social, and mother/child indicators.* T*-tests and* χ*^2^ tests will be used to assess mean and proportional differences in both cross-sectional and changes in repeated measures across study time points for participant outcomes. As this is a pilot study, powered analyses are not the purpose of this project and thus we do not have a target number of participants for enrollment [55]. The research team will not be conducting subgroup or interim analyses.

Qualitative data collected during the community needs assessment will be analyzed using the procedure for Rapid Identification of Themes from Audio Recordings (RITA) described by Neal and colleagues [56]. Though the inclusion of qualitative data can greatly enrich quantitative data, the slow and labor-intensive process of analyzing it can be a barrier due to time constraints [57, 58]. The RITA approach was developed as way to increase the speed of the analysis process while preserving more of the original data. It is applied to the actual audio recordings of semi-structured interviews. The process involves several time-saving steps to improve efficiency of qualitative data analysis. These include pinpointing research foci for which RITA will be used, creating a codebook of key themes identified and their valence (positive, neutral, or negative context), creating a coding form, refining the codebook, coding, and analyzing codes. A key time-saving feature of RITA is that during analysis, audio recordings are broken down into shorter time segments. The coding form is a grid listing themes and time segments, and coders mark the themes present in each time segment [56].

### Data management

All data is stored on password-protected computers or in locked cabinets. Participant identifiers are stored separately from the coded participant data, audio recordings will be destroyed at the end of the study. Study data and documentation monitoring occurs monthly throughout the study and is conducted by the principal investigator and nurse care manager. Study findings will be reported via peer-reviewed publication, a final report to the state of Utah, and national presentations.

### Harms

Potential harms to participants are minimal. For providers, minor psychological risks may include feelings of inadequacy if they are not currently following or have insufficient knowledge of current opioid use treatments for pregnant women. Participants who have treated or are currently treating pregnant women with opioid use disorder may experience feelings of guilt if personal biases or stigmas are uncovered towards pregnant women using opioids. There may be a risk of loss of confidentiality. The project leadership team will meet weekly with the nurse care manager to ensure adherence to the protocol when enrolling and treating patient-level participants.

For patients, the baseline and follow up appointments, patients are asked questions about private, personal matters. The assessments ask demographics, medication adherence, substance use and co-morbid conditions such as pain, depression, etc. The risk of harm is the same as regular health care treatment. Some answers given in research visits (like whether patients use illegal drugs or have ever been arrested) might put them at risk if ever there is a breach of privacy; however, we do not anticipate such a breach. There are no known psychological risks associated with the interview questionnaires or procedures in this study. It is possible that discussion of sensitive topics such as substance use may cause emotional discomfort in some participants. There may also be risks of, emotional distress, inconvenience and possible loss of privacy and confidentiality associated with taking part in a research study.

Medical and psychological support is available through local partnering agencies. If medical needs arise that cannot be addressed at a partnering clinic, the patient will be transported to the local hospital. The research team does not anticipate needing to stop the provider or patient interventions. Providers will be encouraged to offer evidence-based treatment practices for all participants, and treatments provided to participants will be tailored to support the patient’s preferences and individual needs. This project does not have an official Data Monitoring and Safety Board; however, the Principal Investigator and research staff will routinely monitor all aspects of the study conduct and documents, and implement corrective actions as any deviations are observed.

## Discussion

This study protocol is of a type 1 hybrid effectiveness-implementation intervention [28] using two implementation frameworks to guide and assess the success of a perinatal OUD education intervention in a rural community. The hybrid type 1 design allows the research team to focus on clinical effectiveness monitoring while also gathering information on the implementation of the intervention [28]. Given the urgent need for identifying methods for implementing evidence-based practices to treat OUD among pregnant women, we chose to use this hybrid effectiveness-implementation design to help disseminate state-of-the-art-treatment as quickly and efficiently as possible [28]. Our hybrid type 1 is specifically examining the intervention at the patient level, while observing the implementation process and barriers.

Combining two implementation frameworks to supplement one another is commonly used in the literature [59–62]. The CFIR is comprehensive regarding the assessment, and the RE-AIM framework is the intervention approach. The CFIR was employed at the beginning of the study to assess for contextual factors that may affect implementation and throughout the project to assess impact. The CFIR is a large implementation framework, and has been employed extensively to assess MOUD interventions [63]. Previous research shows that tailoring the CFIR to focus more on patient needs within the *outer setting* domain improves its utility in outpatient healthcare settings [64]. With this in mind, it may be useful for our research team to focus in more specifically on this domain during implementation of our intervention. The CFIR was also used in a narrative review of 20 published articles to evaluate attitudes towards prescribing BUP [63]. This review found that the domains *intervention characteristics, outer setting,* and *inner setting* were represented in the analysis, and concerns about diversion, self-efficacy in prescribing, philosophical objections, and stigma were identified as top barriers to BUP prescribing. Our project aims to improve provider self-efficacy in caring for pregnant women with OUD and reduce stigma, addressing the barriers identified in the aforementioned study. Knowledge derived from our project regarding barriers to adoption of the intervention have been and will continue to be used to guide the topics of our webinars and other forms of provider education.

Due to the physical distance between the community of interest and the research team, the use of video and telecommunication is key in delivering this intervention. Previous research has adapted the CFIR to assess readiness for and suitability of a tele-mentoring platform, the Project Extension for Community Healthcare Outcomes (ECHO), and created ECHO-specific organizational readiness questions and a process guide for implementation [65]. Using the CFIR as a guide for our project may also yield information that can be adapted and utilized as guidelines for other interventions involving OUD-specific remote education.

The RE-AIM framework was chosen to evaluate the implementation of this provider education intervention because RE-AIM is an established tool for comprehensively assessing multilevel interventions and contains a dimension for assessing efficacy of the intervention itself (Table 2) [27]. The RE-AIM evaluation framework may also be useful in implementing an intervention similar to ours on a larger scale. This framework was utilized by McNeely and colleagues to rigorously evaluate an OUD treatment consult model in a large multi-site trial, generating knowledge for how OUD treatment practices can be widely disseminated and adopted across healthcare systems [59]. Similarly, we may be able to apply our RE-AIM evaluation findings to larger scale interventions.

This study is being conducted in the midst of an ongoing opioid crisis in the US, [66] which is increasingly affecting pregnant women and women of child-bearing age [67]. Rural pregnant women and their infants have been severely affected as a group, [68] yet there is a gap in research around evidence-based treatment for perinatal OUD. By measuring treatment outcomes of MOUD specifically among pregnant women, this study is contributing to an emerging knowledge base, which can be utilized to help address the issue of OUD within this high-need population. Specifically, our intervention may benefit this population by providing wrap-around services through the support of a care manager, and enhancing quality of prenatal care by training community providers in best evidence-based practices for perinatal OUD and NOWS treatment. The findings derived from this study will benefit the field at large by providing evidence for models of care for pregnant women struggling with OUD, and informing implementation of similar interventions across the country.

### Future directions

This hybrid effectiveness-implementation study has the potential to improve health outcomes for pregnant women with OUD and their infants, and to inform perinatal OUD treatment practices in rural communities across the country. Next steps for this work include implementation with other rural communities in the greater US struggling with problem of perinatal OUD. This may include reaching out to county and state governments, as well as national organizations for collaborative partnerships to support similar programs implementing perinatal OUD education to community healthcare providers. Qualitative findings from this study identifying needs and barriers may also be used to drive future program development and research.

## Limitations

While this study has several strengths including its focus on implementation of evidence-based care, targeting an underserved population within rural communities, and the inclusion of providers, its limitations should be discussed. This implementation intervention was designed for a specific rural community, which could potentially limit generalizability of the findings; however, rural counties across the US appear to be experiencing similar challenges, [5] and this intervention may be adapted for site-specific needs. This study is a single arm trial with no control group, and therefore evaluation through an implementation framework, like RE-AIM, is critical in adequately assessing the study’s effect on intervention effectiveness, uptake and sustainability.

## Conclusion

To our knowledge, this is the first study to specifically implement perinatal OUD treatment education to providers and services to pregnant women in a rural area. Many rural pockets of the Mountain West and other rural areas in the US are disproportionately struggling with the opioid crisis [69]. For this reason, we are implementing a type 1 hybrid effectiveness-implementation project providing perinatal OUD education to healthcare providers and resource enrichment, which allows us to efficiently disseminate evidence-based treatment while also monitoring implementation of the intervention [28]. We adopted two complementary implementation frameworks to guide and evaluate the project: the CFIR to assess contextual features that may influence implementation, [26] and the RE-AIM framework to evaluate the success of the intervention and implementation [27]. The comprehensive approach presented in this protocol offers the potential for reproducibility in other rural communities facing the problem of perinatal OUD.

### Protocol version

Issue date 2 April 2020, version 3.

## Supplementary information

**Additional file 1: Figure 1.** Community Representative Needs Assessment Interview Guide.** Figure 2. **Healthcare Provider Needs Assessment Interview Guide.** Figure 3.** Patient Advocate Needs Assessment Interview Guide.

## Data Availability

Deidentified data from this study will be available upon request from the corresponding author 1 year after all aims of the project are completed.

## Abbreviations

OUD: Opioid use disorder
NOWS: Neonatal opioid withdrawal syndrome
MOUD: Medications for opioid use disorder
CFIR: Consolidated framework for implementation research
RE-AIM: Reach effectiveness adoption implementation maintenance
SBIRT: Screening, brief intervention, and referral to treatment
AUDIT-C: Alcohol use disorders identification test
DSM-5: The diagnostic and statistical manual 5
FTND: Fagerstrom test for nicotine dependence
DAST: Drug abuse screening test
EBPAS: Evidence-based Practice Attitude Scale
EPDS: Edinburgh postnatal depression scale
PHQ-9: Patient health questionnaire-9
GAD-7: Generalized anxiety disorder-7
K6: Kessler Psychological Distress Scale
SF12: 12-Item short form survey
ACE: Adverse childhood experiences
PC-PTSD-5: Primary Care PTSD Screen for DSM-5
PTSD: Post-traumatic stress disorder
PAFAS: Parenting and family adjustment scale
PSQ-18: Patient Satisfaction Questionnaire Short Form
TSR-6: Treatment services review-6
APNCU: Adequacy of prenatal care utilization
HIV: Human immunodeficiency virus
RITA: Rapid identification of themes from audio recordings
BUP: Buprenorphine
ECHO: Project extension for community healthcare outcomes

## References

[1] Haight SC, Ko JY, Tong VT, Bohm MK, Callaghan WM (2018). Opioid Use Disorder Documented at Delivery Hospitalization - United States, 1999–2014. MMWR Morb Mortal Wkly Rep.

[2] Gemmill A, Kiang MV, Alexander MJ (2019). Trends in pregnancy-associated mortality involving opioids in the United States, 2007–2016. Am J Obstet Gynecol.

[3] Smid MC, Stone NM, Baksh L, Debbink MP, Einerson BD, Varner MW (2019). Pregnancy-associated death in utah: contribution of drug-induced deaths. Obstet Gynecol.

[4] Smid MC, Maeda J, Stone NM, Sylvester H, Baksh L, Debbink MP (2020). Standardized criteria for review of perinatal suicides and accidental drug-related deaths. Obstet Gynecol..

[5] Brown JD, Goodin AJ, Talbert JC (2018). Rural and appalachian disparities in neonatal abstinence syndrome incidence and access to opioid abuse treatment. J Rural Health.

[6] ACOG Committee Opinion No (2012). 524: Opioid abuse, dependence, and addiction in pregnancy. Obstet Gynecol.

[7] Jones HE, Heil SH, Baewert A, Arria AM, Kaltenbach K, Martin PR (2012). Buprenorphine treatment of opioid-dependent pregnant women: a comprehensive review. J Addict.

[8] McQueen KA, Murphy-Oikonen J, Gerlach K, Montelpare W (2011). The impact of infant feeding method on neonatal abstinence scores of methadone-exposed infants. Adv Neonatal Care.

[9] Shainker SA, Saia K, Lee-Parritz A (2012). Opioid addiction in pregnancy. Obstet Gynecol Surv.

[10] Whiteman VE, Salemi JL, Mogos MF, Cain MA, Aliyu MH, Salihu HMJJop. Maternal opioid drug use during pregnancy and its impact on perinatal morbidity, mortality, and the costs of medical care in the United States. 2014;2014. 10.1155/2014/906723 PMC4164310 25254116

[11] Schiff DM, Nielsen T, Terplan M, Hood M, Bernson D, Diop H (2018). Fatal and nonfatal overdose among pregnant and postpartum women in Massachusetts. Obstet Gynecol.

[12] Committee Opinion No (2017). 711: opioid use and opioid use disorder in pregnancy. Obstet Gynecol.

[13] Jones HE, Heil SH, Baewert A, Arria AM, Kaltenbach K, Martin PR (2012). Buprenorphine treatment of opioid-dependent pregnant women: a comprehensive review. Addiction.

[14] Keough L, Fantasia HC (2017). Pharmacologic treatment of opioid addiction during pregnancy. Nurs Womens Health.

[15] Haug NA, Duffy M, McCaul ME (2014). Substance abuse treatment services for pregnant women: psychosocial and behavioral approaches. Obstet Gynecol Clin North Am.

[16] Villapiano NL, Winkelman TN, Kozhimannil KB, Davis MM, Patrick SW (2017). Rural and urban differences in neonatal abstinence syndrome and maternal opioid use , 2004 to 2013. JAMA Pediatrics..

[17] Martin CE, Longinaker N, Terplan M (2014). Recent trends in treatment admissions for prescription opioid abuse during pregnancy. J Subst Abuse Treat.

[18] Andrilla CH, Moore TE, Patterson DG, Larson EH (2019). Geographic distribution of providers with a DEA waiver to prescribe buprenorphine for the treatment of opioid use disorder: a 5-year update. J Rural Health..

[19] Sigmon SC (2014). Access to treatment for opioid dependence in rural America: challenges and future directions. JAMA Psychiatry.

[20] Patrick SW, Buntin MB, Martin PR, Scott TA, Dupont W, Richards M (2019). Barriers to accessing treatment for pregnant women with opioid use disorder in Appalachian states. Subst Abus.

[21] Villapiano NL, Winkelman TN, Kozhimannil KB, Davis MM, Patrick SW (2017). Rural and urban differences in neonatal abstinence syndrome and maternal opioid use, 2004 to 2013. JAMA pediatrics.

[22] Krans EE, Patrick SW (2016). Opioid use disorder in pregnancy: health policy and practice in the midst of an epidemic. J Obstetrics Gynecol.

[23] Andrilla CHA, Moore TE, Patterson DG (2019). Overcoming barriers to prescribing buprenorphine for the treatment of opioid use disorder: recommendations from rural physicians. J Rural Health.

[24] Andrilla CHA, Coulthard C, Larson E (2017). Barriers rural physicians face prescribing buprenorphine for opioid use disorder. Ann Fam Med.

[25] Andrilla C, Coulthard C, Larson E (2017). Changes in the supply of physicians with a DEA DATA waiver to prescribe buprenorphine for opioid use disorder.

[26] Damschroder LJ, Aron DC, Keith RE, Kirsh SR, Alexander JA, Lowery JC (2009). Fostering implementation of health services research findings into practice: a consolidated framework for advancing implementation science. Implement Sci.

[27] Glasgow RE, Vogt TM, Boles SM (1999). Evaluating the public health impact of health promotion interventions: the RE-AIM framework. Am J Public Health.

[28] Curran GM, Bauer M, Mittman B, Pyne JM, Stetler C (2012). Effectiveness-implementation hybrid designs: combining elements of clinical effectiveness and implementation research to enhance public health impact. Med Care.

[29] The Henry J Kaiser Family Foundation. Opioid Overdose Deaths by Gender [Report on the Internet]. KFF; 2018. https://www.kff.org/other/state-indicator/opioid-overdose-deaths-by-gender/?dataView=1¤tTimeframe=0&sortModel=%7B%22colId%22:%22Female%22,%22sort%22:%22desc%22%7D. Accessed 2 May 2019.

[30] Desai RJ, Hernandez-Diaz S, Bateman BT, Huybrechts KF (2014). Increase in prescription opioid use during pregnancy among Medicaid-enrolled women. Obstetrics Gynecol..

[31] RHIhub. Am I Rural? [Report on Internet]. RHI; 2019 https://www.ruralhealthinfo.org/am-i-rural/report?lat=39.55136&lng=-110.38588&addr=84539%2C%20UT&exact=0. Accessed 4 Nov 2019

[32] Vandenack T (2019). Ogden ranks high in opioid overdose deaths, trails just Carbon, Emery counties. Standard-Examiner.

[33] Murphy K. How Carbon County is 'building a fence' to protect the community from opioids. Deseret News. 2017; Sect. InDepth.

[34] Fondario A. Opioid Overdose Crisis in Carbon County. Department of Health; 2016.

[35] Callan LB (1971). Adapting the windshield survey model to community health education. HSMHA Health Rep.

[36] CFIR Research Team. Ann Abor: CFIR Guide. CFIR Domains; 2019. https://cfirwiki.net/guide/app/index.html#/guide_select. Accessed 4 Nov 2019.

[37] Aarons G (2004). Mental health provider attitudes toward adoption of evidence-based practice: the evidence-based practice attitude scale. Mental Health Serv Res.

[38] Venkat A, Aldridge A, Kearney S, Radack J, Richard-Aasen S, Grasso K (2017). Derivation of a shortened research instrument for measuring alcohol and other drug (AOD) attitudes in a screening, brief intervention, and referral to treatment (SBIRT) training program. J Sci, Human Arts..

[39] Anderson P, Clement S (1987). The AAPPQ revisited: the measurement of general practitioners' attitudes to alcohol problems. Br J Addict.

[40] Dawson DA, Grant BF, Stinson FS, Zhou Y (2005). Effectiveness of the derived Alcohol Use Disorders Identification Test (AUDIT-C) in screening for alcohol use disorders and risk drinking in the US general population. Alcohol Clin Exp Res.

[41] Tarrahi MJ, Rahimi-Movaghar A, Zeraati H, Motevalian SA, Amin-Esmaeili M, Hajebi A (2015). Latent class analysis of DSM-5 criteria for opioid use disorders: Results from the Iranian National Survey on Mental Health. Eur Addict Res.

[42] Pomerleau CS, Carton SM, Lutzke ML, Flessland KA, Pomerleau OF (1994). Reliability of the fagerstrom tolerance questionnaire and the fagerstrom test for nicotine dependence. Addict Behav.

[43] Yudko E, Lozhkina O, Fouts A (2007). A comprehensive review of the psychometric properties of the drug abuse screening test. J Subst Abuse Treat.

[44] Cox JL, Holden JM, Sagovsky R (1987). Detection of postnatal depression development of the 10-item edinburgh postnatal depression scale. Br J Psychiatry..

[45] Kroenke K, Spitzer RL, Williams JB (2001). The PHQ-9: validity of a brief depression severity measure. J Gen Intern Med.

[46] Lowe B, Decker O, Muller S, Brahler E, Schellberg D, Herzog W (2008). Validation and standardization of the Generalized Anxiety Disorder Screener (GAD-7) in the general population. Med Care.

[47] Staples LG, Dear BF, Gandy M, Fogliati V, Fogliati R, Karin E (2019). Psychometric properties and clinical utility of brief measures of depression, anxiety, and general distress: The PHQ-2, GAD-2, and K-6. Gen Hosp Psychiatry.

[48] Ware J, Kosinski M, Keller SD (1996). A 12-Item Short-Form Health Survey: construction of scales and preliminary tests of reliability and validity. Med Care.

[49] Felitti VJ, Anda RF, Nordenberg D, Williamson DF, Spitz AM, Edwards V (1998). Relationship of childhood abuse and household dysfunction to many of the leading causes of death in adults. The Adverse Childhood Experiences (ACE) Study. Am J Prev Med..

[50] Prins A, Bovin MJ, Smolenski DJ, Marx BP, Kimerling R, Jenkins-Guarnieri MA (2016). The primary care PTSD screen for DSM-5 (PC-PTSD-5): development and evaluation within a veteran primary care sample. J Gen Intern Med.

[51] Sanders MR, Morawska A, Haslam DM, Filus A, Fletcher R (2014). Parenting and family adjustment scales (PAFAS): validation of a brief parent-report measure for use in assessment of parenting skills and family relationships. Child Psychiatry Hum Dev.

[52] Thayaparan AJ, Mahdi E (2013). The patient satisfaction questionnaire short form (PSQ-18) as an adaptable, reliable, and validated tool for use in various settings. Med Educ Online.

[53] Cacciola JS, Alterman AI, Lynch KG, Martin JM, Beauchamp ML, McLellan AT (2008). Initial reliability and validity studies of the revised Treatment Services Review (TSR-6). Drug Alcohol Depend.

[54] Finnegan LP, Connaughton JF, Kron RE, Emich JP (1975). Neonatal abstinence syndrome: assessment and management. Addict Dis.

[55] Bacchetti P (2002). Peer review of statistics in medical research: the other problem. BMJ (Clinical research ed).

[56] Neal JW, Neal ZP, Vandyke E, Kornbluh M (2015). Expediting the analysis of qualitative data in evaluation: a procedure for the rapid identification of themes from audio recordings (RITA). Am J Evaluat.

[57] Halcomb EJ, Davidson PM (2006). Is verbatim transcription of interview data always necessary?. Appl Nurs Res.

[58] Tessier S (2012). From field notes, to transcripts, to tape recordings: evolution or combination?. Int J Qualitat Methods.

[59] McNeely J, Troxel AB, Kunins HV, Shelley D, Lee JD, Walley A (2019). Study protocol for a pragmatic trial of the consult for addiction treatment and care in hospitals (CATCH) model for engaging patients in opioid use disorder treatment. Addict Sci Clin Pract.

[60] Tabak RG, Schwarz CD, Kemner A, Schechtman KB, Steger-May K, Byrth V (2019). Disseminating and implementing a lifestyle-based healthy weight program for mothers in a national organization: a study protocol for a cluster randomized trial. Implement Sci.

[61] King DK, Shoup JA, Raebel MA, Anderson CB, Wagner NM, Ritzwoller DP (2020). Planning for implementation success using re-aim and cfir frameworks: a qualitative study. Front Pub Health.

[62] Arrossi S, Paolino M, Orellana L, Thouyaret L, Kohler RE, Viswanath K (2019). Mixed-methods approach to evaluate an mHealth intervention to increase adherence to triage of human papillomavirus-positive women who have performed self-collection (the ATICA study): study protocol for a hybrid type I cluster randomized effectiveness-implementation trial. Trials.

[63] Louie DL, Assefa MT, McGovern MP (2019). Attitudes of primary care physicians toward prescribing buprenorphine: a narrative review. BMC Fam Pract.

[64] Safaeinili N, Brown-Johnson C, Shaw JG, Mahoney M, Winget M (2020). CFIR simplified: pragmatic application of and adaptations to the consolidated framework for implementation research (CFIR) for evaluation of a patient-centered care transformation within a learning health system. Learn Health Syst.

[65] Serhal E, Arena A, Sockalingam S, Mohri L, Crawford A (2018). Adapting the consolidated framework for implementation research to create organizational readiness and implementation tools for project ECHO. J Cont Educat Health Profess.

[66] Lyden J, Binswanger IA (2019). The United States opioid epidemic. Semin Perinatol.

[67] Terplan MJ (2017). Women and the opioid crisis: historical context and public health solutions. Fertility Sterility.

[68] Stockwell S (2017). Rural pregnant women and newborns hit hard by opioid crisis. Am J Nurs.

[69] Rigg KK, Monnat SM, Chavez MN (2018). Opioid-related mortality in rural America: Geographic heterogeneity and intervention strategies. Int J Drug Policy.

