# Supplementary material for: Addressing opioid use disorder among rural pregnant and postpartum women: a study protocol
Source: Addict Sci Clin Pract. 2020 Oct 31;15:33. doi: 10.1186/s13722-020-00206-6 (PMC7603672; doi:10.1186/s13722-020-00206-6)
Supplement: Supplementary file 1 — Additional file 1: Figure 1. Community Representative Needs Assessment Interview Guide. Figure 2. Healthcare Provider Needs Assessment Interview Guide. Figure 3. Patient Advocate Needs Assessment Interview Guide. [file 13722_2020_206_MOESM1_ESM.docx]

**Additional file 1: Figure 1. Community Representative Needs Assessment Interview Guide**

| **Community Representative Questions** |
| --- |
| Can you describe from your perspective how severe is the problem in this community of pregnant women who have opioid use disorder (OUD) and their children not having appropriate care or services? |
| What kinds of conversations or other forms of communication have you had with community members about this problem? |
| What kinds of communication have you had with other people outside of your counties? |
| In general, how much do you interact or communicate with your peers outside of your counties about the problem of opioid use among pregnant women in your community? |
| Tell me about any times that your organization encouraged you to network with other communities to help pregnant women with OUD. |
| Would you please tell me about any programs or services you are aware of that treat pregnant women with OUD in your community? |
| How about in surrounding communities? |
| Just as a reminder, the goal of our project is to partner with the community here in your county in order to improve treatment and health outcomes for pregnant women with opioid use disorder and their children. We’re planning to do this by offering education for healthcare providers on best treatment practices for these women and their children, and also by leveraging funds from the state into the community for services like care management, behavioral health services, and alternative therapies, like yoga and acupuncture. |
| How do you think bringing our program into your counties will be helpful? |
| Are there any barriers that you can think of that will make it difficult for us to conduct this project? |
| Is there anything else you would like us to know about your community? |

**Additional file 1: Figure 2. Healthcare Provider Needs Assessment Interview Guide**

| **Healthcare Provider Questions** |
| --- |
| Can you describe from your perspective how severe is the problem in this community of pregnant women who have opioid use disorder (OUD) and their children not having appropriate care or services? |
| In terms of developing an integrated solution for OUD among pregnant women and their children, how far along in your opinion is the community and service system in coming up with and implementing a plan? |
| In thinking about working together with us and other community members in developing a solution for OUD among pregnant women, what do you see as the biggest barriers for you and/or the community? |
| I'm going to ask you a hypothetical question. Imagine you went to sleep tonight and woke up tomorrow morning and the problem of pregnant women and their children being affected by the opioid crisis was being handled perfectly. What would that look like? |
| Thanks for sharing that with me. Will you tell me a little bit more about your clinical practice with pregnant women with OUD and/or their children? |
| Prompts: |
| ·       What are some strengths? |
| ·       What are some challenges? |
| ·       How long have you had a practice with either of these populations? |
|  |
| What kind of support do you get from other people inside and outside of your clinic when it comes to treating pregnant women with OUD and their kids? |
| What are some changes you would like to see to improve treatment for pregnant women with OUD and/or their children? |
| Just as a reminder, the goal of our project is to partner with the community here in your counties in order to improve treatment and health outcomes for pregnant women with opioid use disorder and their children. We’re planning to do this by offering education for healthcare providers on best treatment practices for these women and their children, and also by leveraging funds from the state into the community for services like care management, behavioral health services, and alternative therapies, like yoga and acupuncture. |
| Now that I know a little bit more about your clinic and the changes that would be helpful, would you mind sharing your thoughts with me about the project we’re planning to get started in your community? |
| Does the project seem feasible to you? |
| What would make it more feasible? |
| What about this project do you think will be effective? |
| Is there anything else you would like to share with us? |

**Additional file 1: Figure 3. Patient Advocate Needs Assessment Interview Guide**

| **Patient Advocate Questions** |
| --- |
| Can you describe from your perspective how severe is the problem in this community of pregnant women who have opioid use disorder (OUD) and their children not having appropriate care or services? |
| What benefits are there to bringing services like medication-assisted treatments and special treatment for infants experiencing opioid withdrawal to your counties? |
| Do you think pregnant women with OUD in your community will engage in the program? Why or why not? |
| Can you describe some barriers that might come up for women trying to join our program? |
| Just as a reminder, the goal of our project is to partner with the community here in your counties in order to improve treatment and health outcomes for pregnant women with opioid use disorder and their children. We’re planning to do this by offering education for healthcare providers on best treatment practices for these women and their children, and also by leveraging funds from the state into the community for services like care management, behavioral health services, and alternative therapies, like yoga and acupuncture. |
| Does anything about the program that we are planning seem too complex or unclear? |
| Could you tell me about some of the treatment options that are currently available for pregnant women struggling with OUD? |
| How does the program that we plan to bring here compare to other programs or services you know about to treat pregnant women with OUD in your community? |
| Do you feel like pregnant women with OUD in your community are ready to use the program we're hoping to bring through this project? |
| Is there anything else you would like to share with us? |
